# Extended Reconstruction of the Right Forearm as Treatment for a Giant Cell Tumor With the Combined Use of a Free Fibular Flap and a Pedicled Inguinal Flap

**DOI:** 10.7759/cureus.95393

**Published:** 2025-10-25

**Authors:** Guillermo Sergio Dorantes-Millan, Rodrigo Banegas Ruiz, Ana Claudia López Contreras, Jesús Rigoberto Covarrubias Rodríguez, Alejandro Elnecave Olaiz, Francisco Emilio Ferreira Aparicio, Gerardo Rafael Gutiérrez Sevilla

**Affiliations:** 1 Plastic Surgery, Hospital General Dr. Fernando Quiroz Gutierrez, Mexico City, MEX; 2 Plastic Surgery, Department of Plastic and Reconstructive Surgery, Instituto Nacional de Rehabilitación Luis Guillermo Ibarra Ibarra, Mexico City, MEX; 3 Plastic and Reconstructive Surgery Residency Program, National Autonomous University of Mexico (UNAM), Mexico City, MEX; 4 Plastic Surgery, Specialized Burn Unit, Centro Nacional de Investigación y Atención de Quemados (CENIAQ), Mexico City, MEX; 5 Medical Education and Orthopedic Surgery, Instituto Nacional de Rehabilitación Luis Guillermo Ibarra Ibarra, Mexico City, MEX

**Keywords:** bone and soft tissue reconstruction, flap, free fibular flap, microsurgical reconstruction, reconstructive surgical procedures

## Abstract

Giant cell tumor of bone (GCTB) is a benign neoplasm with aggressive behavior, predominantly affecting young adults. In the majority of these lesions, curettage is the treatment of choice; however, those classified as Campanacci grade III typically require extensive surgical resection, resulting in significant bone and soft tissue defects. This represents a significant reconstructive challenge but offers the opportunity for limb preservation and functional maintenance instead of amputation.

We present a 39-year-old male with a grade III GCTB of the distal radius, who had extensive tumor resection followed by a staged reconstructive approach in four distinct surgical phases. These involved excision of the lesion, reconstruction with a free fibular flap, and skin coverage using a pedicled inguinal flap. The postoperative course was uneventful, with no evidence of recurrence at three months. Functional recovery was favorable, with a wrist range of motion of 40° flexion and 30° extension. No postoperative complications were observed.

The combined use of a free fibular flap and a pedicled inguinal flap proved to be an effective reconstructive strategy for managing complex upper extremity circumferential defects.

## Introduction

Giant cell tumor of bone (GCTB) is a benign primary bone tumor with a high tendency for local recurrence following curettage. Although malignant transformation is rare, it may occur. The tumor derives its name from the abundance of reactive multinucleated giant cells observed microscopically. These giant cells contribute to bone resorption; however, they are not true neoplastic cells. Instead, the stromal mononuclear cells are believed to constitute the actual neoplastic component, driving the recruitment and formation of multinucleated giant cells. These giant cells resemble osteoclasts both morphologically and functionally. They may originate from the fusion of monocyte/macrophage lineage cells, similar to foreign-body giant cells seen in chronic inflammation. Regardless of their exact lineage, their activity contributes to the osteolytic lesions characteristic of this tumor [1].

GCTB accounts for approximately 15-20% of all benign bone tumors and about 4-5% of all bone tumors. It primarily affects individuals aged 20 to 45 years [1].

Pain is undoubtedly the most frequent symptom experienced by these patients. Additional symptoms depend on the tumor’s size and involvement of adjacent joints or nerves. These may include swelling, restricted range of motion, paresis, paresthesia, and, in cases where the cortical bone is affected, pathological fractures [1,2].

For the staging and radiographic grading of the tumor, imaging tests are crucial. These tumors typically have radiological borders that are quite clearly defined, clinically active, and limited to the bone. Lesions can be staged using the Campanacci grading method based on the radiograph's results. Grade I lesions are limited to the bone. Grade II lesions are those that feature an enlargement of the cortex but no perforation, and grade III lesions are those that have soft tissue extension and cortical perforation. Based on the extent of local disease, various surgical modalities are used for definitive management. Surgical options can vary from intralesional curettage to wide local excision [1,2].

Surgical intervention continues to be the only curative treatment available for this disease, the type of surgery is determined by the degree of Campanacci and the location of the tumor, curettage and adjuvant therapy are preferable in tumors with a grade I and II; however, a grade III is treated more aggressively with extensive excision because they have a high risk of recurrence, Radical resection is primarily indicated in cases involving substantial bone loss where simple resection is insufficient; however, these procedures require extensive reconstructive efforts to preserve limb function. This is why it is increasingly necessary to explore reconstructive options and seek the one that brings the best benefits to our patients [2,3].

It is well-established that the resection of bone tumors in the extremities often results in large bone and soft tissue defects. While the topic remains debated, it is crucial to consider limb salvage over amputation whenever feasible, as functional outcomes are generally superior with limb preservation compared to those achievable with prosthetic devices. Bone reconstruction can be performed using non-vascularized bone grafts for defects no larger than 5 cm and surrounded by healthy tissue. Cases that do not meet these criteria or are considered hostile are better managed with vascularized bone flaps, which have demonstrated improved outcomes [3].

The free fibular flap has become the gold standard for bone reconstruction due to its advantages, including a long vascular pedicle, capacity for hypertrophy, ease of harvest, and high rates of successful integration [3].

For soft tissue defects in the upper extremities, the McGregor flap is a reliable pedicled flap harvested from the inguinal region, also named the groin flap, and based on the superficial circumflex iliac artery. Its versatility allows for effective coverage of defects ranging from the forearm to the elbow, including extensive circumferential skin losses. The flap’s robust vascular supply and relative ease of harvest make it an excellent option for reconstructing complex soft tissue defects in this anatomical region [4].

Indications for a single fibular flap include upper extremity reconstruction involving both bone and skin defects, as well as reconstruction of bone defects in regions subjected to relatively low mechanical stress [5].

## Case presentation

This is the case of a male patient, 39 years old, right-hand dominant, a farmer, with no relevant medical history, who presented for evaluation due to a history of progressive tumor growth in the right forearm over the past eight years. He reported direct trauma to the distal third of the right forearm in May 2015, after which he noted the appearance of a 1 × 1 cm mass that resolved spontaneously without associated symptoms.

Subsequently, in January 2017, the patient was involved in a motor vehicle accident with direct trauma to the same region. This was followed by the appearance of a second tumor, which exhibited progressive growth but remained asymptomatic. He was evaluated by a private physician, who performed an incisional biopsy. The histopathological report confirmed a diagnosis of giant cell tumor (GCT). No treatment was initiated at that time.

**Figure 1:**
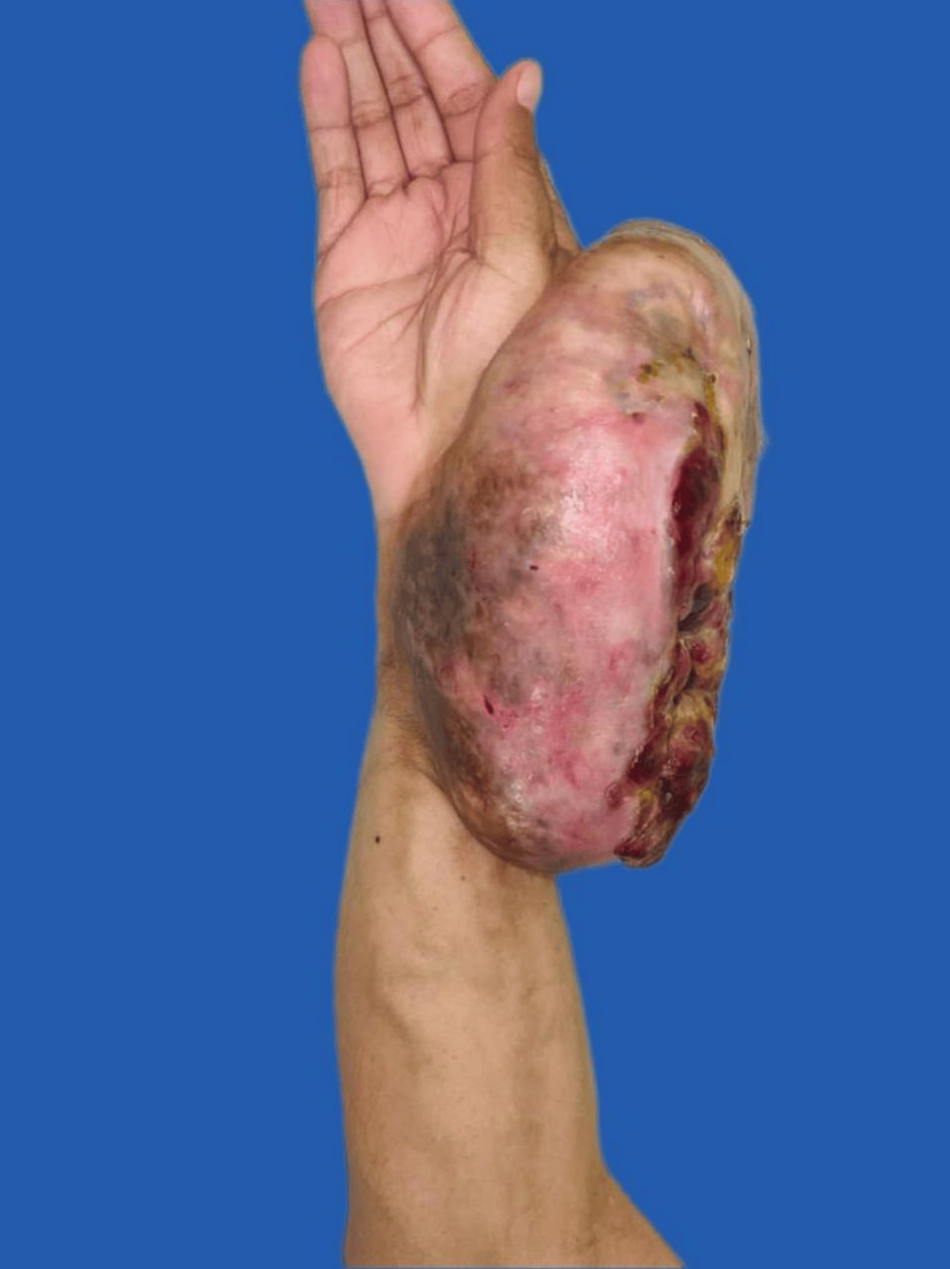
Clinical Image of the right forearm with an ulcerated tumor lesion in volar view

Due to continued tumor growth, the patient returned for medical evaluation in April 2023. On physical examination, a tumor was observed on the right upper extremity, involving both the dorsal and volar aspects of the forearm, measuring approximately 30 × 15 cm. The mass was of stony consistency, with superficial neovascularization and a 10 × 6 cm ulcer with irregular borders, without active bleeding, but with purulent discharge and necrotic tissue. Distal mobility and sensation in the fingers were preserved, with a capillary refill time of two seconds (Figure 1).

**Figure 2:**
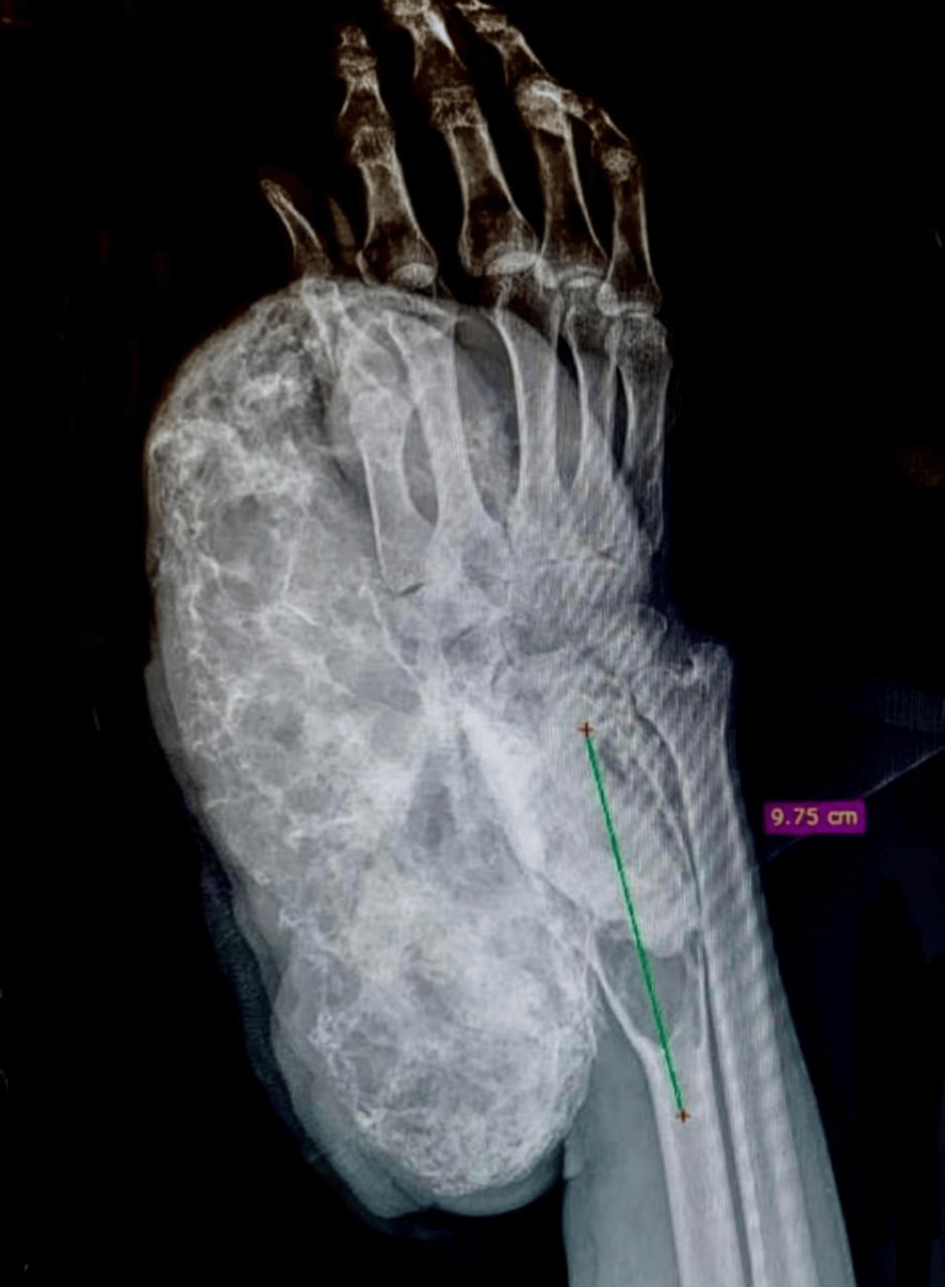
Radiograph of the right forearm in anteroposterior projection with an expansive bone lesion that involves the distal radius

A preoperative diagnostic protocol was initiated. Anteroposterior, lateral, and oblique radiographs of the right wrist revealed a cystic lesion in the distal metaphysis of the radius, communicating with an extensive lytic area measuring 22.86 × 13.09 cm. The lesion extended from the middle third of the radius to the carpal region, with involvement of the scaphoid and lunate bones, as well as disruption of both the anterior and posterior cortices of the radius (Figure 2).

**Figure 3:**
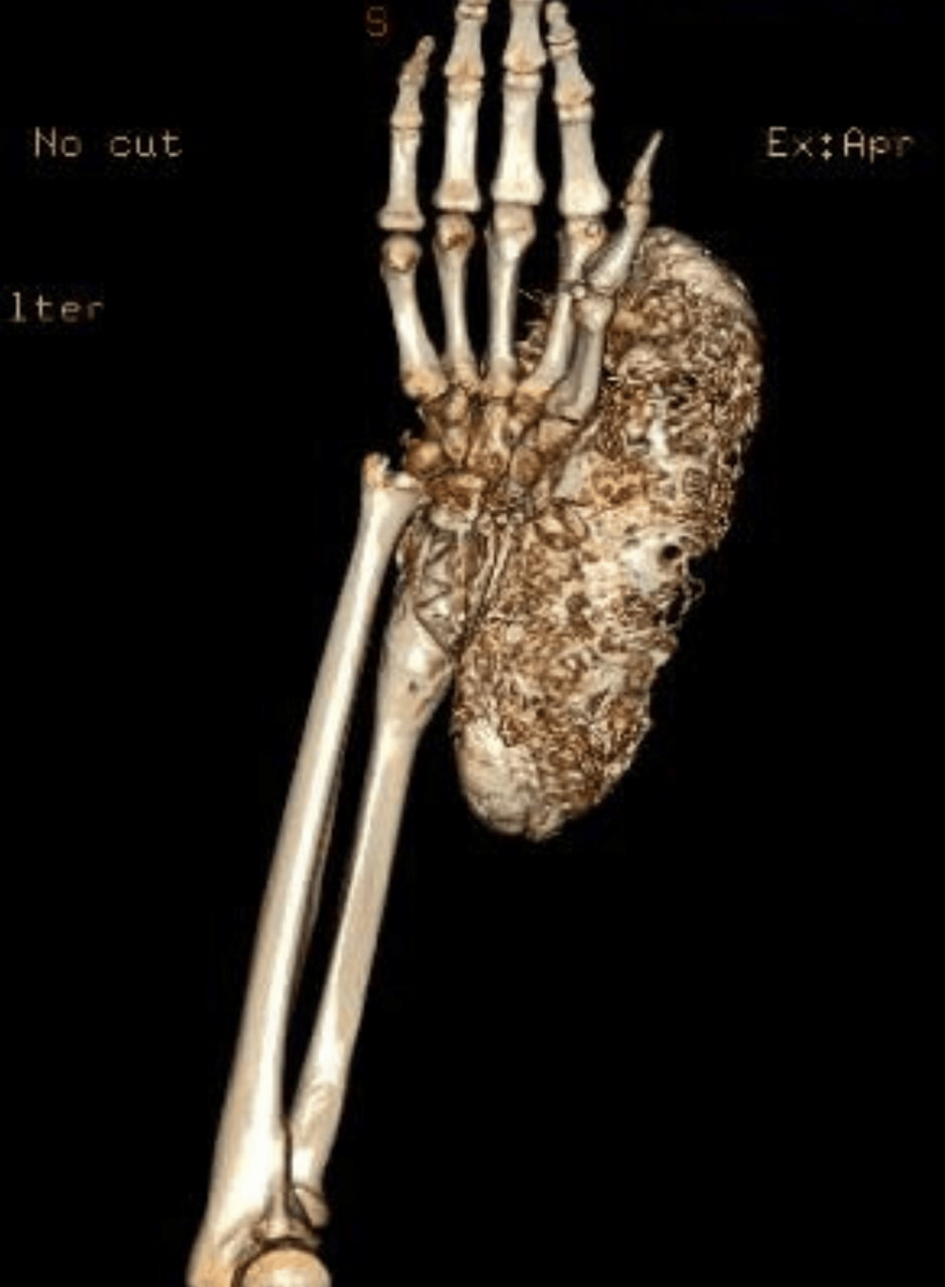
Extensive osteolytic lesion in the distal radius in a three-dimensional reconstruction tomography of the right forearm

Computed tomography of the wrist demonstrated a lytic lesion in the metaepiphyseal region of the distal radius, with attenuation values of 33 Hounsfield Units (HU), non-sclerotic margins, widening of the transition zone, and cortical breach on the lateral aspect. There was an extension into the subchondral bone of the scaphoid, lunate, and the head of the ulna. The lesion, measuring approximately 20 × 11 × 10 cm, exhibited soft tissue invasion, mass effect, and the presence of air locules with fistulous tracts extending to the skin surface, resulting in dermal irregularity (Figure 3).

The staging protocol was completed with chest, pelvis, and skull radiographs, revealing no evidence of metastatic disease. Laboratory tests were within normal limits. Based on the clinical presentation, imaging findings, and histopathological results, a decision was made to proceed with staged surgical treatment, taking into account the extent of the bone and soft tissue defects.

**Figure 4:**
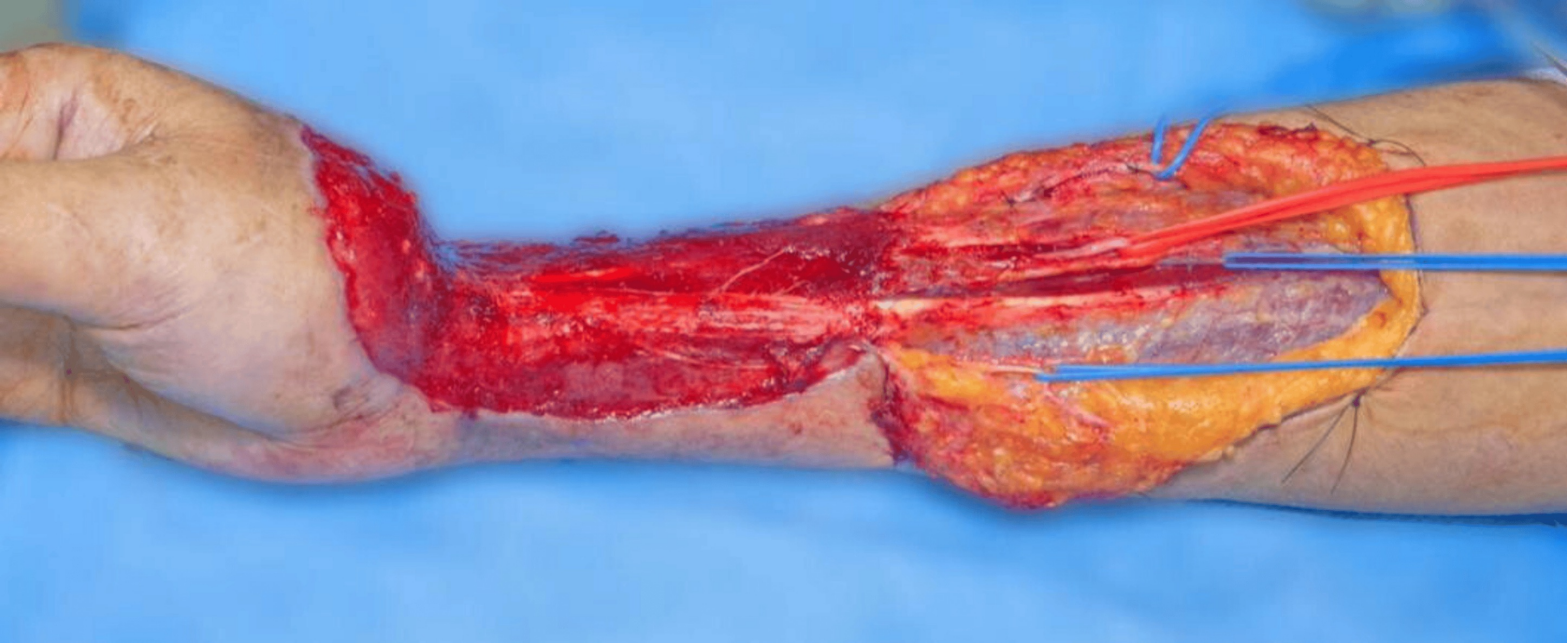
Intraoperative image of the right forearm after tumor resection, showing the extensive lesion area in the distal radius

The first surgical procedure was performed following proper preparations with asepsis and antisepsis of the region, and the application of sterile fields. An Esmarch bandage was placed to induce ischemia in the right upper limb. A circumferential approach to the tumor was carried out, with dissection according to the anatomical planes. It was noted that the tendons of the first, second, and third dorsal compartments of the wrist, as well as the radial nerve and artery, were involved in the tumor. A bone osteotomy was performed approximately 4 cm proximal to the macroscopic origin of the lesion. The involved structures were carefully dissected and released from the tumor. The surgical specimen was then removed (Figure 4). A 3.0 Steinmann nail was placed from the fourth metacarpal to the ulna for stabilization. Ischemia was reversed, hemostasis was confirmed, and a negative-pressure wound therapy system was applied. The surgical procedure was completed without complications.

The specimen was analyzed by pathology, with the following results.

Macroscopic description: Surgical specimen was 22 cm long x 12 cm wide and 7 cm thick, with multiple areas of ulceration in the skin. The specimen, upon sectioning, demonstrated a lesion with numerous cystic formations containing both purulent and hemorrhagic material.

**Figure 5:**
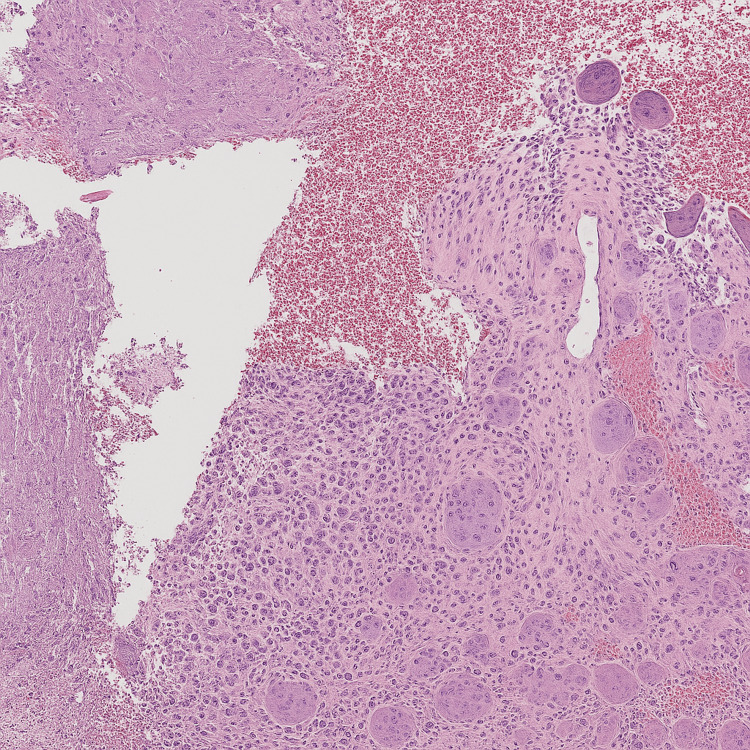
Histopathological image showing the lesion composed of multinucleated giant cells interspersed with stromal cells, with areas of recent hemorrhage

Microscopic description: The lesion was composed of multinucleated giant cells interspersed with stromal cells (Figure 5).

Final diagnosis: Histopathology indicated a giant cell tumor.

The patient remained under negative pressure wound therapy for surgical bed preparation. The second stage of surgery was scheduled, with a diagnosis of a large bone and skin circumferential defect of the right forearm, secondary to sequelae of giant cell tumor of the right radius, Campanacci classification grade 3.

The patient was taken to the operating room for the scheduled procedure under standard monitoring and surgical protocols. The negative pressure wound therapy system (VAC), which had been in place for seven days, was removed from the right thoracic extremity. A proximal extension of the skin defect was performed to improve surgical exposure. The radial artery was identified, showing a significant decrease in flow. The adventitia of the artery was dissected without a significant improvement. Intraluminal irrigation was performed with 250 mL of physiological solution containing 10,000 units of unfractionated heparin and 50 mL of 2% lidocaine, achieving an improvement in arterial flow. Subsequently, a Fogarty Fr #4 catheter was placed in the artery, advancing it to the 30 cm mark. Saline solution (0.5 mL) was infiltrated to distend the balloon, which was subsequently deflated and removed along with the catheter; a significant improvement in arterial flow was observed. The vascular structures (arteries and veins) were referenced using color-coded Surgiloops: red for arteries and blue for veins. The proximal extension of the surgical wound in the right forearm was closed with simple stitches using 4-0 nylon. A negative pressure system (VAC) was applied over the surgical bed, with no evidence of leakage. In the left lower limb, ischemia was applied at the level of the proximal third of the thigh. An extensive approach was performed to expose the perforating branches of the peroneal artery, which were initially observed to be adequate. Dissection of the soft tissues was carried out until the diaphysis of the fibula was fully exposed. Bone disection was performed, followed by proximal and distal osteotomies, matching the length of the bony defect in the right forearm. Bleeding and the integrity of the bone surface were verified. At this stage, neither the fibula nor the pedicled flap was yet autonomized. Layered closure was performed using 4-0 nylon for deep planes and skin staples for the superficial layer. The tourniquet was released, and hemostasis was verified. Surgical wounds of the lower limb were covered with sterile dressings and elastic bandages. The procedure was completed without complications.

**Figure 6:**
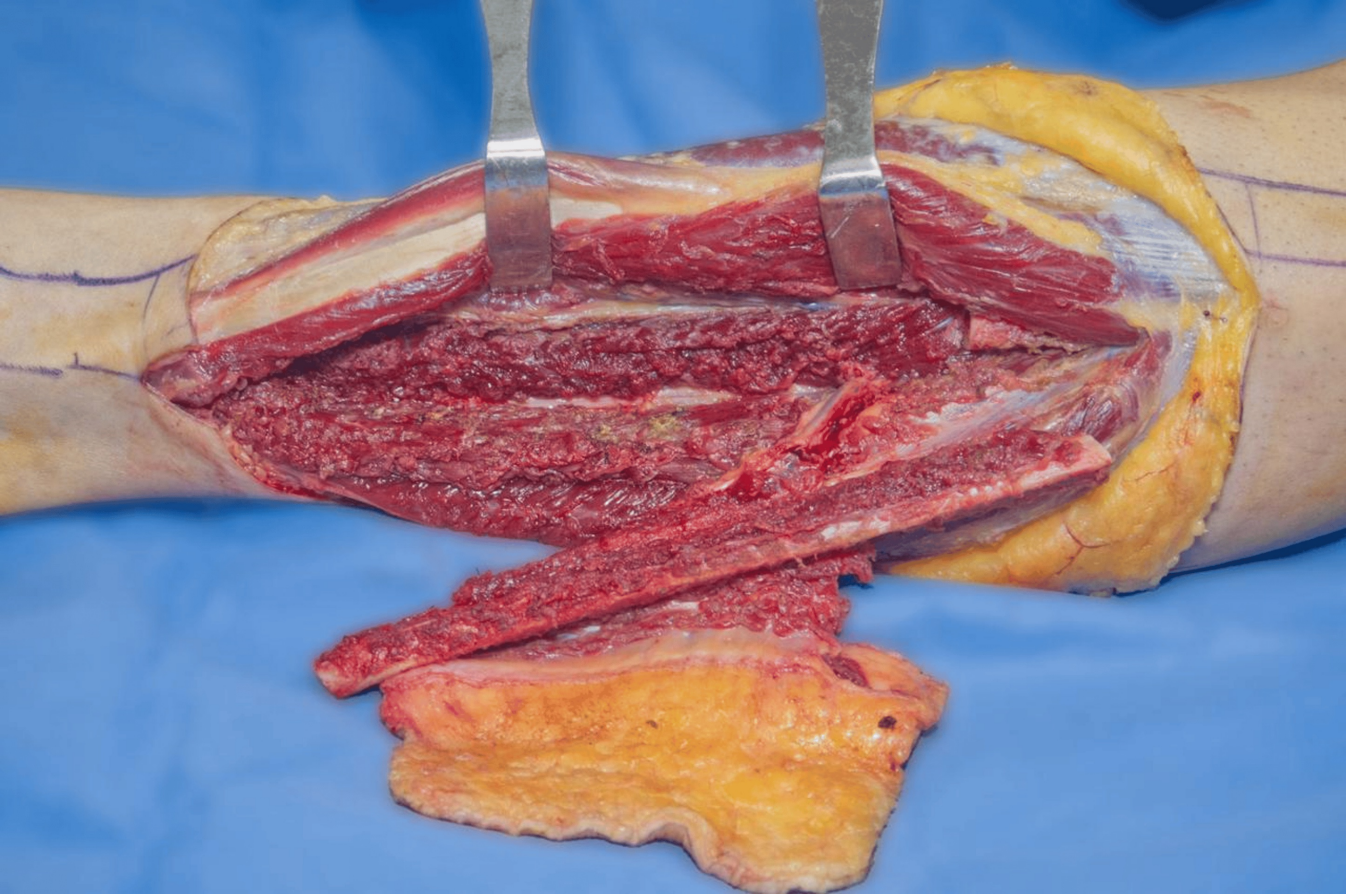
Intraoperative image of the left leg (lateral view): dissection of the fibula to obtain the vascularized bone flap

The patient was scheduled for the third stage of reconstructive treatment, which included removal of the negative pressure system (VAC) and Steinmann nail, transfer of a vascularized bone flap from the fibula to the distal radius, wrist arthrodesis, coverage of the right forearm with an ilioinguinal pedicled flap, and skin graft from the left thigh to the donor region in the left leg. The procedure began with inspection of the flap in the left lower limb, which showed signs of viability. The fibula and its vascular pedicle were identified, ligated, and divided (Figure 6). The area was irrigated, and muscle and fascia plasty was performed.

**Figure 7:**
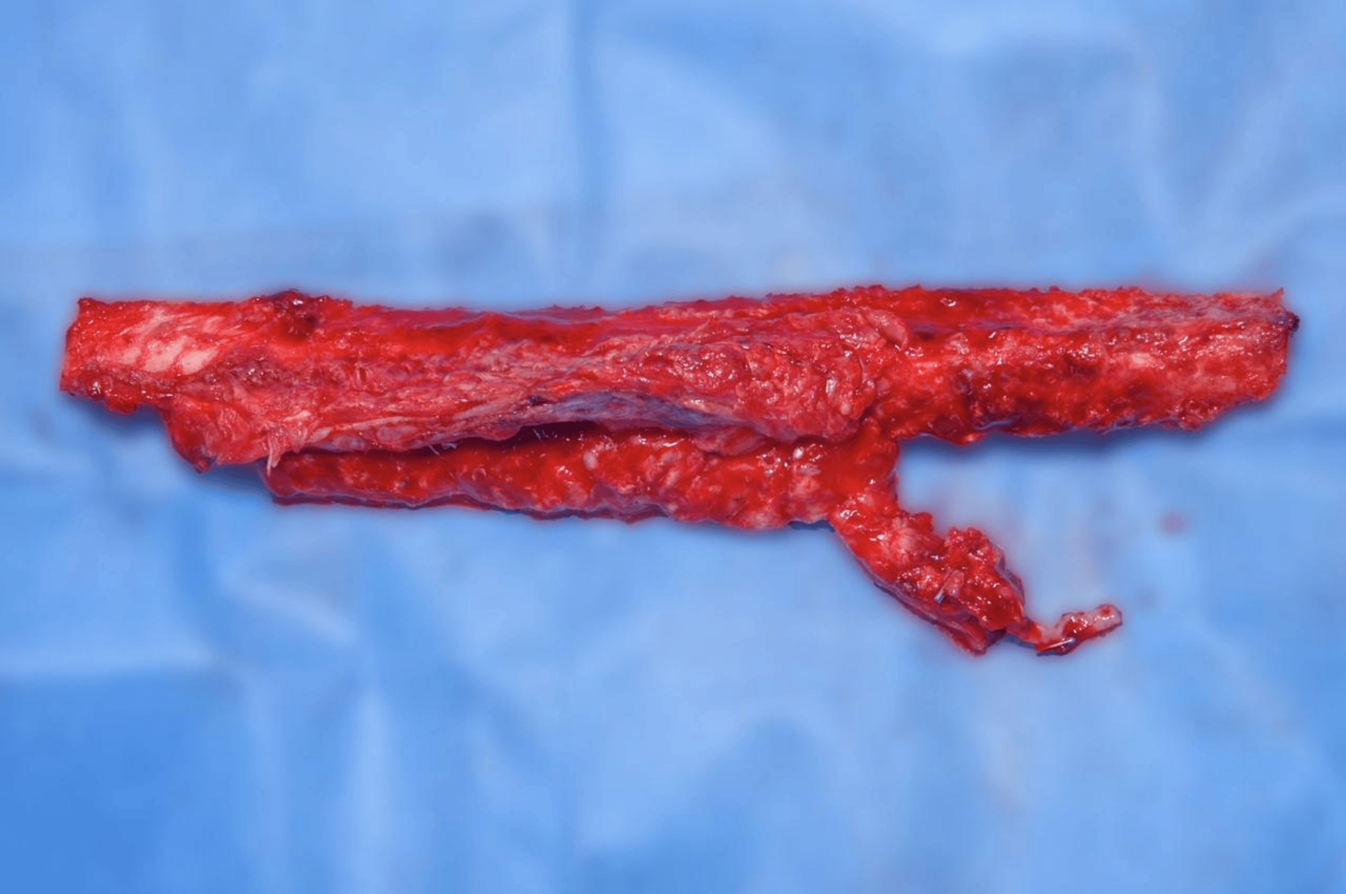
11 cm length of the free fibular flap

Subsequently, the free fibular flap was transferred to the distal region of the right forearm (Figure 7). Osteotomy was performed to achieve adequate congruence with the proximal radius (approximately 11 cm). Layered dissection was performed, the Steinmann nail was removed, and wrist arthrodesis was performed using an anatomic plate attached to the third metacarpal.

The fibula flap was transferred and fixed to the radial diaphysis using a fixation plate. Intraoperative radiographic control was taken, showing adequate placement of the osteosynthetic material and satisfactory bone alignment.

**Figure 8:**
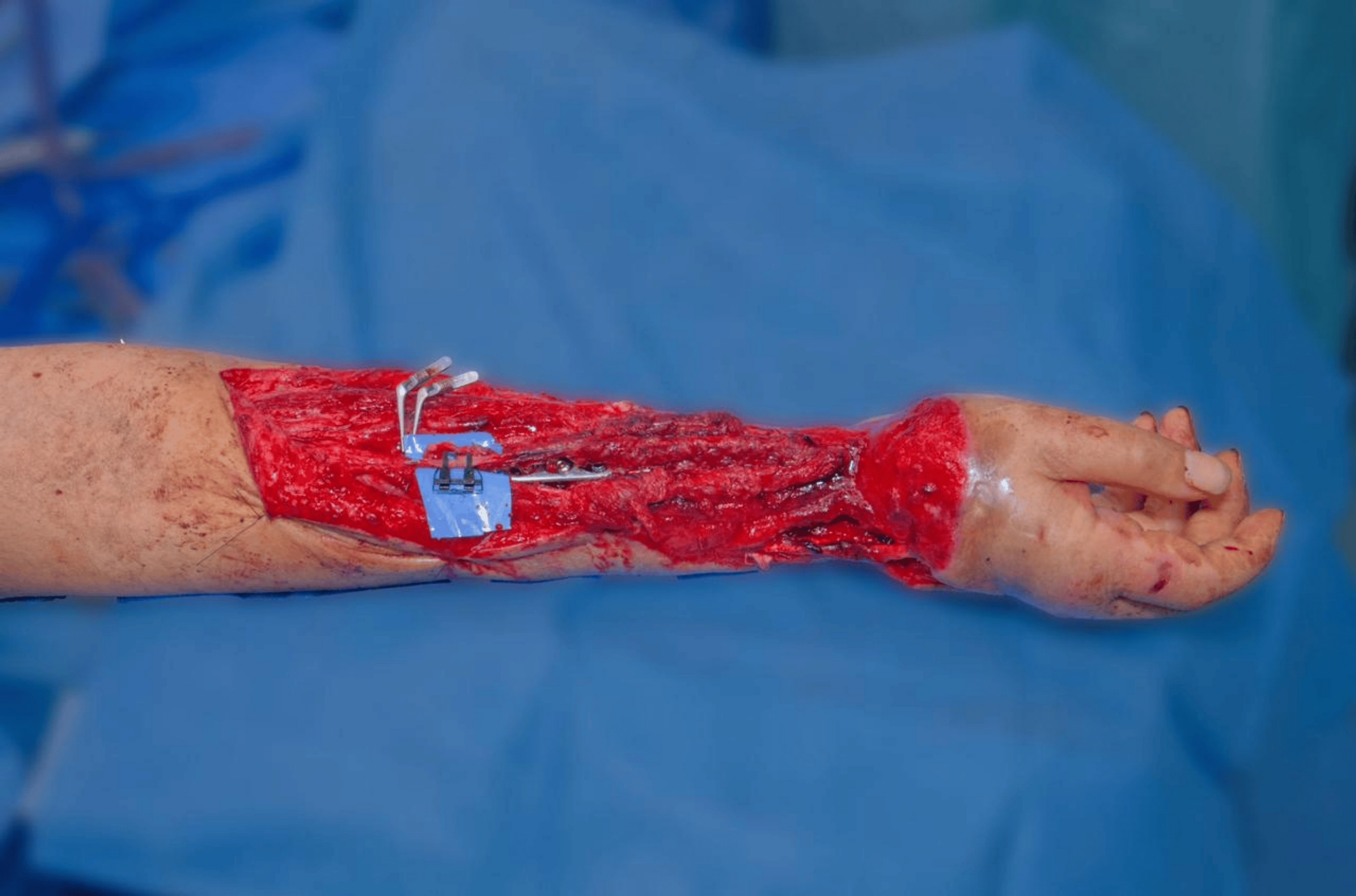
Microvascular anastomosis between the peroneal artery of the flap and the radial artery of the forearm

Microsurgical anastomosis of the vascular structures was performed between the peroneal artery of the flap and the radial artery of the forearm in an end-to-end fashion using 8-0 nylon sutures. Adequate perfusion, good vascular filling, and absence of leakage were observed intraoperatively (Figure 8).

**Figure 9:**
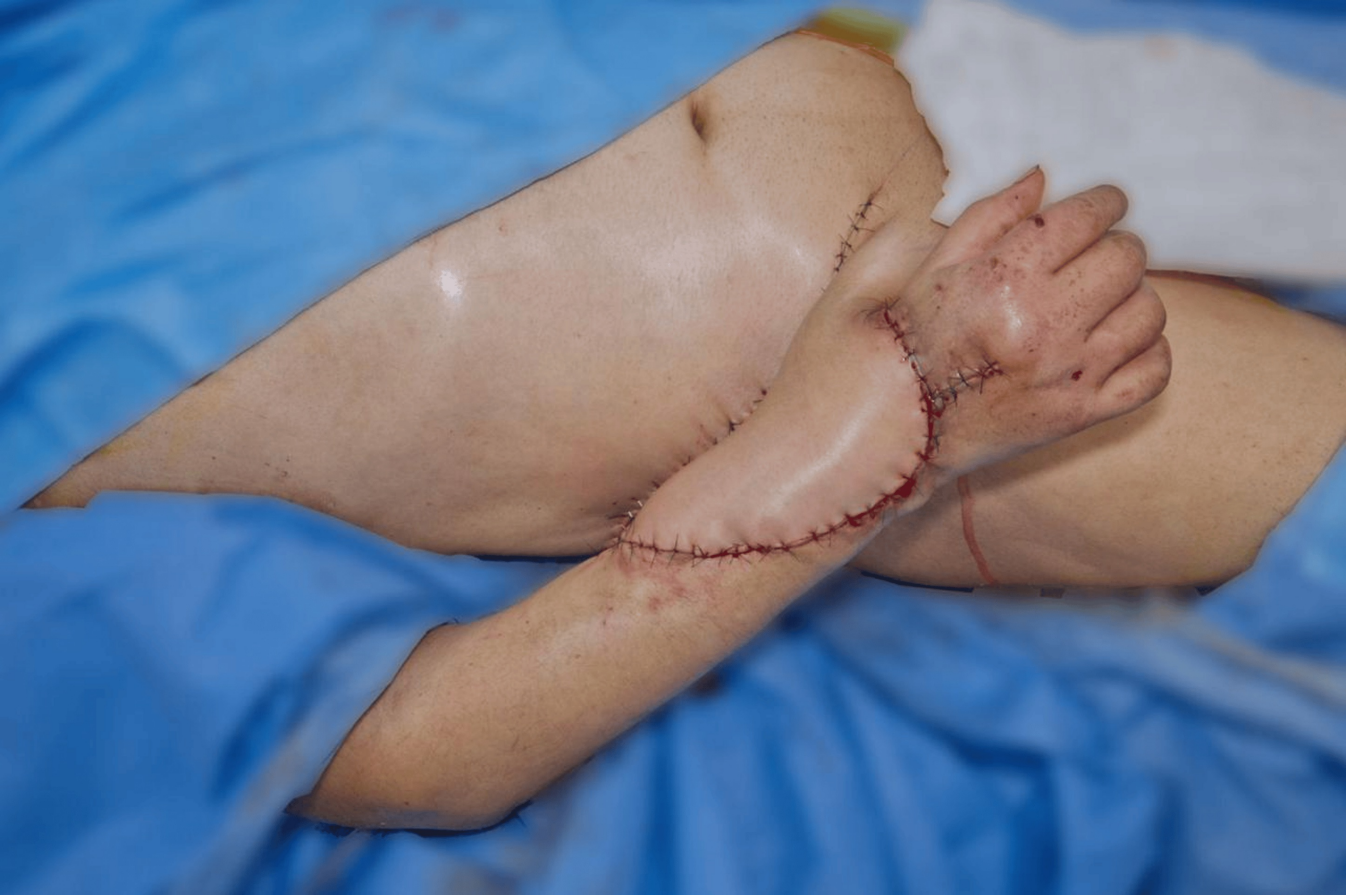
Pedicled ilioinguinal flap (McGregor) installed over the distal defect of the right forearm

Subsequently, an ilioinguinal pedicled flap was created. Following anatomical marking in the abdominopelvic region, a scalpel incision was made, and subcutaneous tissue dissection was carried out. The flap was elevated, and the donor site edges were approximated. The flap was transferred to the right forearm and secured using 3-0 nylon sutures. The flap appeared viable with good perfusion (Figure 9).

Hemostasis was verified, with no evidence of active bleeding. The surgical wounds were irrigated, covered with sterile dressings, and the procedure was concluded without complications.

**Figure 10:**
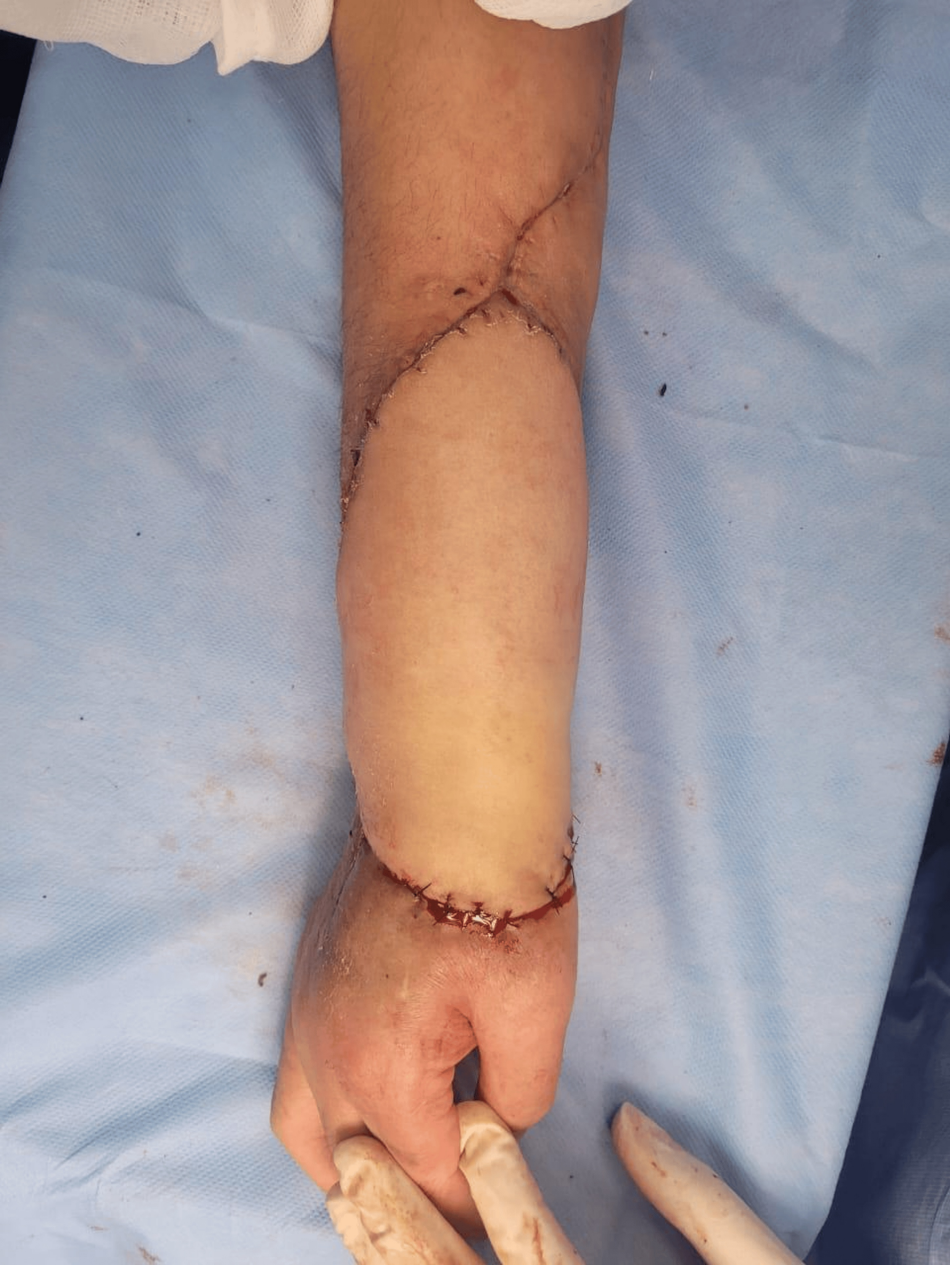
Autonomization and remodeling of the McGregor ilioinguinal flap

After 21 days of adequate postoperative evolution, the patient was scheduled for autonomization and remodeling of the McGregor ilioinguinal flap. Once again in the operating room, and after following proper surgical preparation, a scalpel incision was made along the surgical markings, proceeding with layered dissection. The vascular structures of the flap were located without injuring them, and the dissection continued until the flap was completely freed (Figure 10).

The ilioinguinal donor site was remodeled and closed with 3-0 nylon sutures. Similarly, the flap on the right forearm was remodeled and sutured with 3-0 nylon. Neurostimulation was performed, yielding a satisfactory response. Finally, the area was covered with a sterile dressing and a simple bandage with a window for flap monitoring.

**Figure 11:**
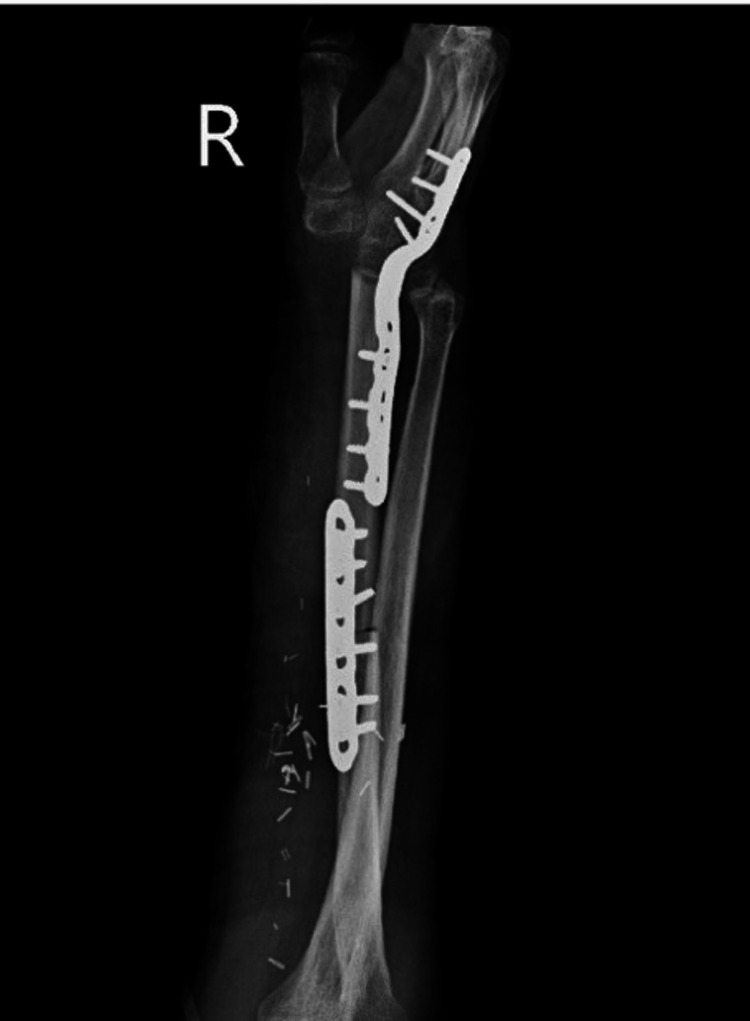
Immediate postoperative radiograph of the forearm demonstrating reconstruction with a vascularized fibular flap, stabilized using a fixation system.

Postoperatively, a radiograph was taken to evaluate the proper fixation of the stabilization system (Figure 11). The patient was monitored for five days, after which he was discharged and referred to the outpatient clinic for continued follow-up by the Rehabilitation Medicine, Bone Tumors, and Plastic and Reconstructive Surgery departments to assess postoperative, aesthetic, and functional progress.

**Figure 12:**
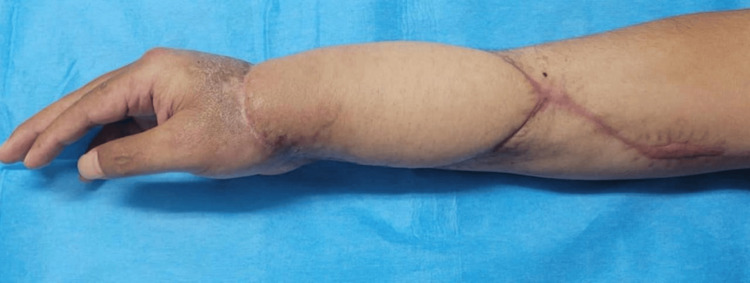
Clinical result at month three (lateral view of the right forearm): healed flap with residual scarring

At the three-month follow-up, the patient was evaluated, with preservation of strength (5/5) and flexion-extension movements conserved with an adequate flap condition, without evidence of recurrence or any factors hindering favorable recovery (Figure 12).

**Figure 13:**
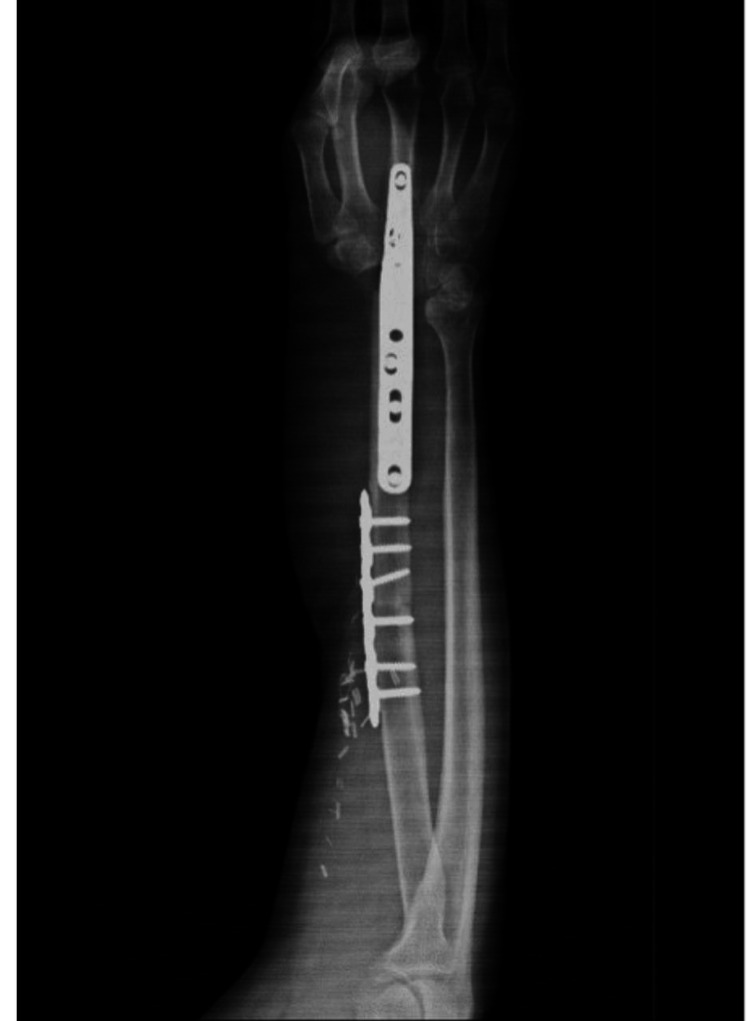
Radiograph at month three showing progressive flap integration with adequate consolidation and maintained stability of the fixation system

A radiograph was taken, showing flap integration with adequate consolidation (Figure 13).

## Discussion

Free fibular flaps currently represent one of the most effective techniques for reconstructing long bone defects in the extremities, particularly following tumor resection. Their usage has increased significantly due to their clinical advantages and favorable post-surgical outcomes.

In cases of primary bone tumors, functional limb reconstruction after radical resection has become possible thanks to surgical advances such as microvascular flaps. Some of these techniques are relatively recent and offer optimal functional outcomes, increasingly regarded as the ‘gold standard’. In the present case, considering the patient’s clinical history and lesion progression, the vascularized peroneal flap was chosen as the most advantageous option. Vascularized bone flaps provide biological benefits by maintaining blood supply to the transplanted tissue, which supports nutrient delivery to deep structures, promotes stable bone healing, and facilitates early mobilization and functional recovery [6,7].

The fibula flap offers ideal length, geometrical shape, and mechanical strength, making it the best donor bone for large bone defects. It is known that free fibular flaps achieve a higher union rate than non-vascularized flaps in bone defects; however, most studies have focused on the bony union rate or the functional outcome of the recipient site after the fibular flap. In a series of bone defects treated by vascularized fibular flaps, bone consolidation was reported to be obtained in 86% to 95% of cases, at a mean of 3.6 to 12 months, significantly outperforming non-vascularized flaps, whose rate is around 63% in similar defects [8].

Additionally, the free fibula flap has adaptive hypertrophy. To be more specific, it can increase its thickness in response to postoperative biomechanical stress, which favors functionality and long-term stability. Its anatomical versatility is also highlighted, as it can be combined with another flap when it is necessary to have a wide cutaneous coverage. Regarding donor site morbidity, reported rates range from 7% to 35%, with complications including pain, limb instability, and neuroma formation. Nonetheless, this technique consistently demonstrates a high consolidation rate and favorable clinical outcomes [7-9].

However, in a comparative analysis, Vyas et al. confirmed that the use of fibular flaps allows for earlier and more efficient consolidation; furthermore, it decreases the number of additional procedures required to achieve bony fusion and recover the functionality of the limb [9].

## Conclusions

Currently, the reconstruction of bone defects secondary to both trauma and oncological resection of aggressive tumors, as in this case, the giant cell tumor, requires meticulous surgical planning and a comprehensive reconstructive approach, because it seeks more than just the amputation of the affected area, the preservation of the affected limb, as well as its functionality. The combination of a free fibular flap and a McGregor pedicled flap represents one of the most complete and effective solutions currently available for large bone and skin circumferential defects. The fibula graft offers not only the possibility of structural restoration thanks to its resemblance to the affected area, as well as its mechanical resistance and capacity for hypertrophy, but also biological integration superior to other grafts. Actually, it preserves the vascular supply, which favors a faster and more stable bone consolidation, especially in defects produced by oncological pathologies. On the other hand, the McGregor type flap provides us with a wide, reliable, and technically accessible skin coverage, ideal to protect both the graft and the underlying structures, especially in cases of severe soft tissue compromise.

In this case, the approach taken not only showed us the efficacy with which both procedures can be combined but also allowed the preservation of the limb, which, in addition to the resection of the tumor, was the initial goal. The approach and techniques used provided progressive functional recovery and a favorable postoperative evolution, with no evidence so far of tumor recurrence or complications in the short term. It is important to mention that for this patient, the approach and outcome could have been different if the treatment had been instituted in the early stages of the disease; even so, it underlines the importance of multidisciplinary intervention in the treatment of advanced bone tumors, including orthopedic oncology, reconstructive surgery, and early rehabilitation, to achieve optimal oncologic and functional results. In our experience, the combined use of the free fibula flap and McGregor flap should be considered a highly effective and reproducible strategy for the reconstruction of complex bone and skin circumferential defects in the upper extremity, with great potential to become a therapeutic standard in selected cases. The success of reconstruction depends not only on surgical technique and bone consolidation but also on a comprehensive postoperative rehabilitation program. Early mobilization, facilitated by stable osteosynthesis and adequate vascularization, significantly contributes to functional recovery by minimizing joint stiffness and muscle atrophy. A multidisciplinary approach is essential to optimize limb function and improve the patient’s quality of life. Together, these factors enable patients to regain independence and return to daily activities.
